# The Invisible Barrier: A Scoping Review of Stigma and Nursing Attitudes in Chemsex Care

**DOI:** 10.3390/nursrep16070227

**Published:** 2026-06-30

**Authors:** Emerson Lucas Junio Silva Camargo, Álvaro Francisco Lopes de Sousa, Alice Silva Costa, Anderson Reis de Sousa, Vinicius de Lima Lovadini, Inês Fronteira, Herica Emilia Felix de Carvalho, Liliane Moretti Carneiro, Carla Aparecida Arena Ventura

**Affiliations:** 1 Ribeirão Preto School of Nursing, University of São Paulo, Ribeirão Preto 14040-902, SP, Brazil; caaventu@eerp.usp.br; 2 Campus de Três Lagoas, Federal University of Mato Grosso do Sul, Três Lagoas 79070-900, MS, Brazil; sousa.alvaromd@gmail.com (Á.F.L.d.S.); liliane.m.carneiro@ufms.br (L.M.C.); 3 NOVA National School of Public Health, Public Health Research Centre, Comprehensive Health Research Center (CHRC), REAL, NOVA University of Lisbon, 1050-091 Lisbon, Portugal; ines.fronteira@ensp.unl.pt; 4 Multiprofessional Program in Family Health, Federal University of Alfenas–UNIFAL, Alfenas 37130-001, MG, Brazil; alice34.trabalho@gmail.com; 5 School of Nursing, Federal University of Bahia, Salvador 40170-110, BA, Brazil; areisconsultor@gmail.com; 6 School of Medicine, Universidade Brasil, Fernandópolis 15600-000, SP, Brazil; viniciuslovadini@hotmail.com; 7 Department of Nursing, State University of Maranhão, Coroatá 65055-310, MA, Brazil; hericacarvalho@professor.uema.br

**Keywords:** chemsex, nurses, stigma, attitudes

## Abstract

**Background:** Chemsex, or sexualized drug use, exists along a continuum ranging from non-problematic, consensual recreational practice across diverse populations to problematic behaviors linked with clinical vulnerabilities, substance dependence, or compulsive disorders. Within nursing practice, understanding this spectrum is essential to mitigate healthcare-related stigma. **Objective:** To map and synthesize evidence on stigma and attitudes among nurses regarding chemsex, identifying implications for practice and research. **Methods:** A scoping review was conducted following the Joanna Briggs Institute methodology and PRISMA-ScR guidelines. Searches were performed across PubMed/MEDLINE, Scopus, Web of Science, CINAHL, EMBASE, and LILACS. Studies involving nurses or healthcare teams focused on stigma, attitudes, or related constructs in chemsex care were included. Data underwent descriptive and thematic synthesis. **Results:** Six studies met the inclusion criteria, showing substantial heterogeneity. Only one focused exclusively on nurses. Stigma and attitudes were rarely assessed explicitly, emerging instead as underlying factors influencing clinical practice, communication, and patient engagement. Key themes included the necessity for non-judgmental care, significant gaps in knowledge and training, variability in clinical practice, and the impact of organizational barriers. A schematic representation was developed to illustrate the interrelationships between stigma, knowledge, professional attitudes, and structural factors influencing healthcare practice. **Conclusions:** This review positions stigma as a central mechanism influencing nursing care in chemsex contexts. The findings underscore critical gaps in nursing-specific evidence and emphasize the need for targeted training, validated measurement tools, and integrated care models. Strengthening stigma-informed, patient-centered approaches is essential to improve care delivery and health outcomes for this population.

## 1. Introduction

Chemsex, commonly defined as the intentional use of psychoactive substances to enhance, prolong, or facilitate sexual experiences, encompasses a wide spectrum of behaviors [1,2]. While heavily studied within gay, bisexual, and other men who have sex with men (MSM) networks [1], contemporary literature demonstrates that sexualized drug use is a broader phenomenon present in the general population, including women and heterosexual individuals, often serving as a non-problematic practice to diversify sexual life. Emerging evidence also demonstrates that chemsex practices occur across diverse gender identities and sexual orientations, although the current literature remains predominantly concentrated among MSM populations and specialized sexual health settings [1,2,3].

From a harm reduction perspective, chemsex practice is not inherently pathological; it operates along a dynamic continuum where consensual, low-risk recreational engagement can coexist with, or transition into, problematic patterns characterized by mental health disorders, substance dependence, and compulsive sexual behavior disorders (CSBD). However, healthcare structures frequently over-pathologize the phenomenon, reinforcing an invisible barrier of structural stigma that deters individuals across all demographics from seeking affirmative health counseling [3,4].

Beyond its biomedical implications, chemsex represents a complex and multifaceted phenomenon that intersects sexual health, mental health, and substance use, often within contexts marked by stigma, marginalization, and structural inequities [1,2]. Individuals who engage in chemsex may experience overlapping vulnerabilities, including minority stress, discrimination, and barriers to accessing healthcare services, particularly in contexts marked by sexual and substance-use-related stigma [5,6]. These factors contribute not only to increased health risks but also to delays in seeking care, underreporting of behaviors, and disengagement from healthcare systems [7,8,9].

In this context, healthcare professionals play a critical role in identifying, addressing, and managing health needs related to chemsex [10,11,12]. Among healthcare professionals, nurses occupy a particularly strategic position, as they are frequently at the frontline of care in sexual health, primary care, emergency services, and harm reduction settings [5,6,10]. Despite the central role nurses may play in sexual health, harm reduction, and patient engagement, the extent to which nursing perspectives, attitudes, and experiences related to chemsex have been explored in the literature remains unclear [5,6,11]. This issue is particularly relevant given the longitudinal and relational nature of nursing care across different healthcare settings, including sexual health services, primary care, mental health care, emergency services, and community-based harm reduction programs. In these contexts, nurses are often among the professionals with the greatest continuity of contact with patients, positioning them as important facilitators of communication, therapeutic engagement, health education, and continuity of care. Consequently, understanding how stigma, attitudes, and preparedness may influence nursing practice is essential for strengthening patient-centered and stigma-informed approaches to chemsex-related health needs [5,6,11].

Nursing practice in these contexts involves comprehensive assessment, patient education, counseling, and coordination of care, placing nurses in a unique position to engage individuals who may otherwise remain disconnected from healthcare services [8]. Moreover, the nature of nursing care, frequently characterized by sustained patient contact and holistic approaches, makes nurses key actors in building trust and facilitating disclosure of sensitive behaviors such as chemsex [5,6,11,12].

However, addressing chemsex in clinical practice is inherently complex and requires a set of advanced competencies. These include the ability to navigate sensitive topics related to sexuality and substance use, apply harm reduction principles, and deliver culturally competent, patient-centered care [6,11,12,13,14]. Such demands align with the principles of advanced nursing practice, which emphasize clinical judgment, autonomy, culturally competent care and the management of complex and stigmatized health conditions [11]. In the context of chemsex, advanced nursing practice extends beyond technical knowledge, requiring professionals to engage with patients in a non-judgmental and empathetic manner, while also addressing broader psychosocial determinants of health [5,6].

A critical factor influencing the quality of care in this context is stigma. Stigma related to both substance use and sexual behaviors has been widely documented as a barrier to healthcare access, contributing to delayed care-seeking, reduced disclosure, and negative healthcare experiences [12,13,14]. Within healthcare settings, stigma may manifest through explicit or implicit attitudes, including moral judgment, discomfort, or avoidance when addressing topics perceived as sensitive or socially deviant. These dynamics are particularly relevant in the context of chemsex, which involves behaviors that are often highly stigmatized, including drug use and non-normative sexual practices.

Existing evidence suggests that stigma can significantly shape interactions between healthcare professionals and individuals who engage in chemsex. For example, patients may anticipate or experience judgment from healthcare providers, leading to reluctance in disclosing chemsex-related behaviors and missed opportunities for appropriate care and prevention [12,15,16]. At the same time, professionals themselves may experience uncertainty, discomfort, or lack of preparedness when addressing chemsex, further reinforcing gaps in care delivery. Studies have highlighted the importance of non-judgmental communication, empathy, and trust-building as essential components of effective care in this context [10,15].

Despite the recognized importance of these factors, current evidence remains fragmented and limited [5,6]. Research on chemsex has predominantly focused on epidemiological patterns, risk behaviors, and health outcomes among individuals engaging in chemsex, with comparatively limited attention given to healthcare professionals’ perspectives and even fewer studies specifically addressing nursing practice [5,11,15]. When professionals are included, studies often adopt a broader focus on multidisciplinary teams or service delivery models, rather than examining specific professional groups such as nurses [5,15,17]. Furthermore, while concepts such as stigma, attitudes, and perceptions are frequently discussed, they are rarely measured explicitly or systematically, particularly in relation to nursing practice.

This gap is particularly concerning given the central role of nurses in delivering care to populations affected by chemsex. A limited number of studies have explored aspects of knowledge, practice, and training needs among healthcare professionals, revealing important gaps in preparedness and confidence [5,6,11,17,18]. Additionally, organizational and structural barriers, such as time constraints, lack of protocols, and fragmented services, further complicate the integration of chemsex-related care into routine practice [17].

Although foundational stigma frameworks were developed in earlier decades, they remain highly influential in contemporary health research and continue to inform recent studies addressing stigma in sexual health and substance use. Importantly, stigma emerges across the literature not as a directly measured variable, but as an underlying and pervasive factor influencing both professional attitudes and patient experiences. This suggests that stigma operates at multiple levels, individual, interpersonal, and structural, shaping the dynamics of care in subtle yet significant ways. However, the absence of studies explicitly examining stigma and attitudes among nurses represents a critical gap in the evidence base, limiting the development of targeted interventions, educational programs, and clinical guidelines [3,18,19].

Addressing this gap is essential for advancing nursing practice and improving health outcomes for individuals who engage in chemsex. A clearer understanding of nurses’ knowledge, attitudes, and potential stigmatizing behaviors is necessary to inform the development of training strategies, support tools, and policies that promote equitable and patient-centered care. Furthermore, mapping the existing evidence can help identify priorities for future research, particularly in relation to stigma reduction, professional education, and the implementation of best practices in diverse healthcare settings.

Given the exploratory nature of this topic and the heterogeneity of available evidence, a scoping review is an appropriate methodological approach to map the extent, nature, and characteristics of research in this field. Therefore, this study aims to map the available evidence on stigma and attitudes among nurses in relation to chemsex, identifying key themes, gaps in knowledge, and implications for nursing practice and research.

## 2. Materials and Methods

This scoping review was conducted following the Joanna Briggs Institute (JBI) methodological framework and is reported in accordance with the PRISMA-ScR guidelines [20,21]. The study protocol was prospectively registered on the Open Science Framework (OSF) (10.17605/OSF.IO/TQXP8). Given the exploratory nature of the topic and the heterogeneity of the available evidence, the scoping review methodology was considered the most appropriate approach to map the extent, range, and characteristics of the literature addressing stigma and attitudes among nurses in relation to chemsex.

### 2.1. Review Question and Eligibility Criteria

The review was guided by the following research question: What is known about stigma and attitudes among nurses in relation to chemsex? The objective was to map and synthesize the available evidence on stigma and attitudes among nurses regarding chemsex, as well as to identify key concepts, knowledge gaps, and implications for nursing practice and research.

Eligibility criteria were defined based on the Population–Concept–Context (PCC) framework recommended by JBI [20]. The population of interest included nurses, nursing professionals, nurse practitioners, and, when applicable, healthcare professionals in multidisciplinary settings where findings could be contextualized to nursing practice. The concept encompasses stigma, attitudes, perceptions, beliefs, prejudice, discrimination, biases, and related constructs associated with chemsex or sexualized drug use. Because stigma was rarely measured explicitly in the identified literature, the review also considered implicit manifestations of stigma reflected in narratives related to judgment, discrimination, communication barriers, discomfort, avoidance behaviors, and recommendations for non-judgmental care.

The context was not restricted, including studies conducted in healthcare and community settings such as primary care services, sexual health clinics, emergency services, mental health care, addiction services, and harm reduction programs.

Studies were included if they: (1) addressed chemsex or sexualized drug use; (2) involved nurses, nursing professionals, or healthcare professionals in contexts relevant to nursing practice; and (3) explored stigma, attitudes, perceptions, communication, preparedness, or related constructs through qualitative, quantitative, mixed-methods, or case-based designs.

Publications in English, Portuguese, or Spanish were considered eligible. Editorials, opinion papers, letters, and commentaries without empirical or analytical contributions were excluded. Studies focusing exclusively on individuals engaging in chemsex without involving healthcare professionals were also excluded.

### 2.2. Information Sources and Search Strategy

A comprehensive search strategy was developed based on three conceptual blocks: (1) the phenomenon of interest (chemsex or sexualized drug use), (2) the population (nurses or healthcare professionals), and (3) the construct of interest (stigma, attitudes, or related concepts).

Searches were conducted on 20 May 2026, in the following electronic databases: PubMed/MEDLINE, Scopus, Web of Science, CINAHL, EMBASE, and LILACS (via the Virtual Health Library). In addition, grey literature searches were performed using Google Scholar.

The search strategy combined, when applicable, controlled vocabulary terms (e.g., MeSH terms, Emtree terms, and CINAHL Headings) and free-text keywords adapted to the syntax and indexing systems of each database. Free-text descriptors included terms such as “chemsex”, “sexualized drug use”, “party and play”, “PnP”, “drug use during sex”, “nurse*”, “healthcare professional*”, “clinician*”, “stigma”, “prejudice”, “discrimination”, “bias”, and “attitude*”. Boolean operators (AND, OR), truncation symbols, and field restrictions were applied according to the requirements of each database to optimize both sensitivity and specificity.

Following peer-review recommendations, additional slang and community-based descriptors associated with chemsex practices, including “slamming”, “wired play”, “high and horny”, “chemplay”, and “boosting”, were incorporated into the updated search strategy. These additional terms were included across databases and grey literature searches to enhance the comprehensiveness and cultural sensitivity of the retrieval process. Despite the broader search strategy, no additional eligible studies were identified through these expanded descriptors.

Grey literature was systematically searched using Google Scholar. The first 200 records sorted by relevance were screened using combinations of the main descriptors related to chemsex, healthcare professionals, and stigma. In addition, reverse reference searching was conducted through manual screening of the reference lists of all included studies to identify potentially relevant publications not retrieved during database searches. Records identified through Google Scholar and reverse searching were exported and integrated into the same screening, deduplication, and eligibility assessment workflow applied to database records.

The complete database-specific search strategies, including syntax adaptations, controlled vocabulary terms, free-text descriptors, Boolean operators, search fields, search dates, and records retrieved for each database, are provided in Appendix A Table A1.

### 2.3. Study Selection

All identified records were exported to reference management software and subsequently uploaded to Rayyan software (version 1.7.4) for screening and eligibility assessment. Duplicate records were automatically identified and manually verified prior to screening.

The study selection process was conducted in two stages. First, titles and abstracts were independently screened by two reviewers (E.L.J.S.C. and A.S.C.) according to the predefined eligibility criteria. Subsequently, the full texts of potentially eligible studies were independently assessed by the same reviewers. Discrepancies between reviewers were resolved through discussion and consensus. When disagreements persisted, a third reviewer (Á.F.L.d.S.) was consulted for arbitration. The selection process was documented using a PRISMA-ScR flow diagram [21].

According to the PRISMA 2020 framework, “records” refer to titles and abstracts identified during database and grey literature searches, whereas “reports” refer to the full-text documents retrieved for detailed eligibility assessment [22].

### 2.4. Data Extraction

Data extraction was performed using a standardized data charting form developed by the research team. The instrument was pilot-tested on a subset of studies to ensure consistency, clarity, and completeness prior to formal extraction.

The following information was extracted from each included study: author(s), year of publication, country, study design, study setting, participant characteristics, type of healthcare professional involved, nursing sample size when available, study objectives, instruments used, and key findings related to stigma, attitudes, knowledge, communication, clinical practices, barriers to care, organizational factors, and training needs.

Particular attention was given to identifying evidence related to stigma and attitudes, including both explicitly measured constructs and implicitly reported aspects, such as non-judgmental care, patient-provider interactions, perceived discrimination, communication barriers, and professional discomfort in addressing chemsex-related issues.

### 2.5. Data Synthesis and Analysis

The data were analyzed using a descriptive and thematic synthesis approach consistent with JBI recommendations for scoping reviews [20].

Initially, a descriptive summary of the included studies was conducted to characterize methodological aspects, participant profiles, settings, and thematic focus of the literature. Subsequently, a thematic synthesis was performed to identify recurring patterns, conceptual dimensions, and gaps across the studies.

The synthesis process involved iterative reading of the extracted material, inductive coding of relevant findings, grouping of codes into thematic categories, and development of overarching analytical themes. The analysis particularly focused on dimensions related to: (1) stigma and discrimination, (2) professional attitudes and beliefs, (3) knowledge and preparedness, (4) communication and patient engagement, (5) clinical practices, (6) barriers to care, (7) organizational and structural factors, and (8) training and educational needs.

Findings were synthesized and presented in both tabular and narrative formats to facilitate comprehensive mapping of the evidence and identification of knowledge gaps relevant to nursing practice and future research.

### 2.6. Methodological Quality Appraisal

In accordance with JBI methodological guidance for scoping reviews, a formal methodological quality appraisal or risk of bias assessment was not conducted [20]. The primary purpose of this review was to map the breadth, range, and nature of the available evidence rather than to evaluate intervention effectiveness or generate pooled estimates.

Therefore, studies were included based on their relevance to the review question and contribution to understanding stigma and attitudes in chemsex-related care contexts, irrespective of methodological hierarchy, sample representativeness, or statistical power.

## 3. Results

**Figure 1:**
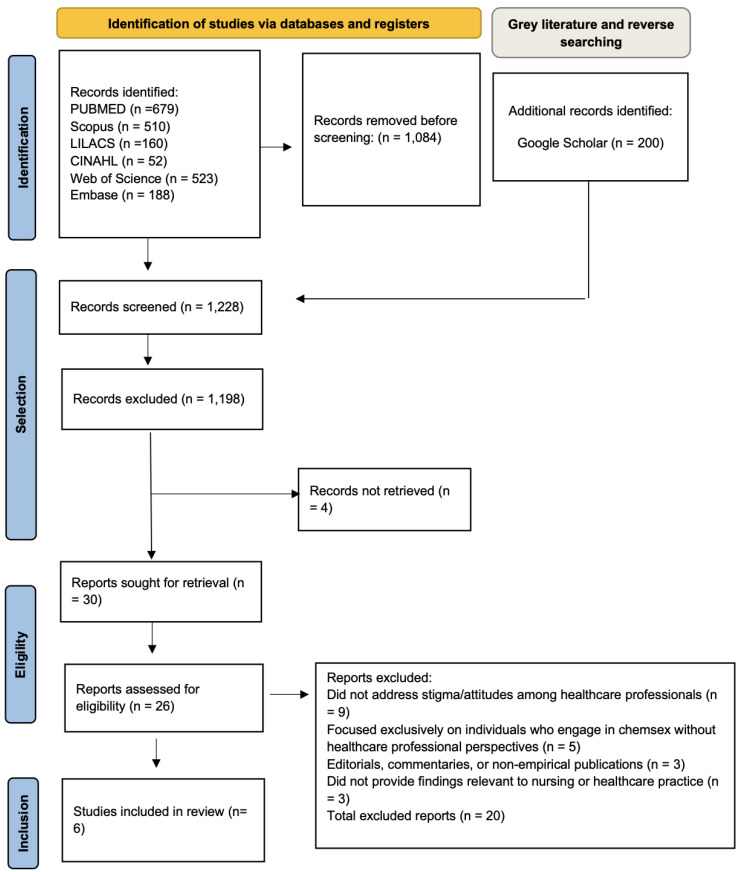
PRISMA 2020 flow diagram of the study selection process [22].

The search process identified a total of 2312 records across electronic databases and grey literature sources. After the removal of 1084 duplicate records, 1228 records remained for title and abstract screening. Following the initial screening process, 30 reports were considered potentially relevant and sought for full-text retrieval, of which four could not be retrieved. A total of 26 full-text reports were assessed for eligibility. After full-text evaluation, 20 reports were excluded for reasons related to the review scope and eligibility criteria, primarily due to the absence of stigma- or attitude-related findings among healthcare professionals or lack of relevance to nursing or healthcare practice. Ultimately, six studies met the inclusion criteria and were included in this scoping review. The study selection process is presented in the PRISMA-ScR flow diagram (Figure 1).

The included studies were published between 2020 and 2026 and demonstrated substantial heterogeneity regarding methodological design, participant composition, and scope of investigation. The evidence base comprised two cross-sectional studies, two qualitative studies, one case-based clinical report, and one professional practice article focused on healthcare responses to chemsex-related issues. Geographically, the studies were conducted across diverse regions, including Europe (Netherlands, United Kingdom, and Italy), North America (United States), and Asia (Thailand), underscoring the international relevance of chemsex as an emerging public health and healthcare concern.

Regarding the populations investigated, only one study focused exclusively on nurses, specifically professionals working in sexually transmitted infection (STI) clinics. The remaining studies involved broader multidisciplinary groups, including nurse practitioners, emergency medical service professionals, harm reduction teams, addiction service professionals, and other healthcare workers involved in sexual health and substance use care. This limited representation of nursing-specific samples highlights an important gap in the literature and reinforces the need for studies that more directly examine nursing perspectives, attitudes, and practices in the context of chemsex-related care.

The characteristics of the included studies are summarized in Table 1. Overall, the studies addressed diverse dimensions of chemsex-related healthcare, including professional knowledge, clinical practices, harm reduction strategies, service organization, communication, and healthcare experiences. However, stigma and professional attitudes were rarely investigated as primary outcomes. Instead, these dimensions emerged indirectly through discussions surrounding patient engagement, non-judgmental care, communication challenges, organizational barriers, and professional preparedness.

The analytical synthesis of the included studies is presented in Table 2. Overall, the evidence reveals a limited and heterogeneous body of research, with stigma and attitudes rarely examined as primary outcomes. Instead, these constructs emerge indirectly, embedded within broader discussions of clinical practice, communication, and barriers to care.

Across the studies, knowledge, attitudes, and clinical practices appear to be closely interconnected, with gaps in training and preparedness influencing professionals’ confidence and approach to chemsex-related issues. Notably, the adoption of non-judgmental and empathetic care is consistently highlighted as essential, reflecting the pervasive role of stigma in shaping both patient engagement and professional behavior.

At the same time, the findings point to inconsistencies in clinical practice and the absence of structured approaches to addressing chemsex, alongside persistent individual and organizational barriers. Importantly, despite the central role of nurses in care delivery, there is a clear lack of studies directly examining stigma and attitudes within nursing, reinforcing a critical gap in the literature.

The thematic synthesis of the findings is presented in Table 3. Overall, the analysis identified a set of recurring and interrelated themes that provide insight into how stigma-related dynamics and professional attitudes may influence the care of individuals who engage in chemsex.

Across the included studies, stigma appeared primarily as an implicit and contextual element rather than as an explicitly measured construct. It was commonly reflected in discussions surrounding communication, patient disclosure, experiences of judgment, and the perceived need for non-judgmental and patient-centered approaches to care. These findings suggest that stigma may play an important role in shaping both patient experiences and professional interactions within chemsex-related care contexts.

The synthesis also indicates that gaps in knowledge, training, and professional preparedness may influence healthcare professionals’ confidence and approaches to addressing chemsex-related issues. At the same time, organizational conditions, including limited resources, fragmented services, and lack of structured protocols, were frequently described as barriers affecting the integration of chemsex-related care into routine practice.

Taken together, the findings suggest that clinical practice in the context of chemsex is shaped by a complex interaction of individual, interpersonal, and structural factors. However, given the limited number of studies, the heterogeneity of methodologies, and the scarcity of nursing-specific evidence, these interpretations should be understood as exploratory rather than definitive. Nevertheless, the identified themes highlight important areas for future research and reinforce the relevance of strengthening competencies related to communication, cultural sensitivity, harm reduction, and stigma-informed care within nursing practice.

The identified research gaps and their implications for both research and nursing practice are summarized in Table 4. Overall, the findings highlight a substantial mismatch between the growing recognition of chemsex as a complex health issue and the limited development of nursing-specific evidence.

Across the included studies, key areas such as stigma, professional attitudes, and knowledge were found to be insufficiently explored, particularly from a nursing perspective. Notably, stigma remains an under-investigated construct, rarely assessed directly and predominantly inferred from patient experiences and recommendations for non-judgmental care. Similarly, attitudes and beliefs of nurses toward chemsex are not systematically examined, limiting the understanding of how these factors influence care delivery.

In addition, important gaps were identified in relation to clinical practice, training, and organizational context. The absence of standardized assessment tools, structured educational interventions, and nursing-specific clinical protocols reflects a broader lack of consolidation in the field. These limitations are further compounded by structural barriers and the limited integration of services, which hinder the implementation of comprehensive and coordinated care.

Taken together, these gaps underscore the need for a more robust research agenda focused on nursing, including the development of validated instruments, evaluation of educational strategies, and investigation of stigma and attitudes as central components of care. From a practice perspective, the findings reinforce the importance of investing in training, organizational support, and the development of integrated care models, positioning nurses as key agents in addressing the complexities of chemsex-related health needs.

**Figure 2:**
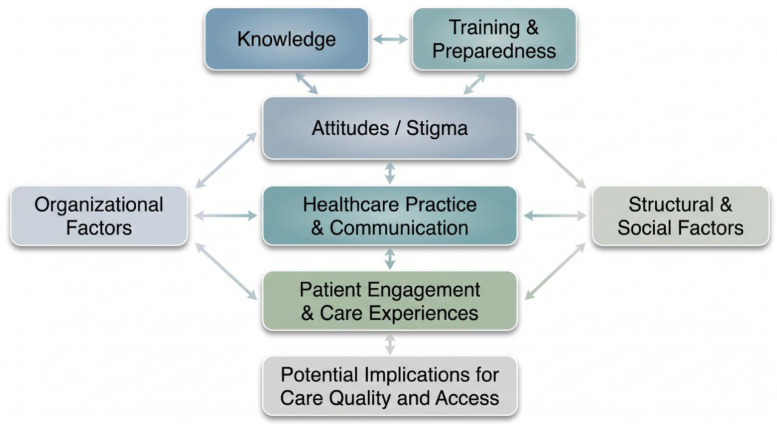
Schematic representation of the interrelated factors identified across the included studies regarding stigma, professional attitudes, and healthcare practice in the context of chemsex.

To further synthesize the relationships identified across the included studies, a schematic representation was developed based on the thematic analysis. The figure illustrates the interconnections between stigma, professional attitudes, healthcare practice, and contextual factors associated with chemsex-related care. The proposed representation is presented in Figure 2.

The schematic representation illustrates the interrelated dimensions identified across the included studies regarding stigma, professional attitudes, and healthcare practice in the context of chemsex. Rather than representing a causal or validated theoretical model, the figure synthesizes the main thematic relationships observed in the literature and highlights how individual, organizational, and structural factors may interact within healthcare settings.

Knowledge and training/preparedness emerged as important elements associated with professional attitudes and stigma-related perceptions. These dimensions were closely connected to healthcare practice and communication, particularly regarding non-judgmental care, patient engagement, and professionals’ confidence in addressing chemsex-related issues. The figure also reflects the influence of organizational and structural/social factors, including service organization, institutional barriers, resource availability, and broader social contexts, which may shape both professional experiences and care delivery practices.

Patient engagement and care experiences are presented as interconnected with healthcare practice and communication, potentially influencing the quality and accessibility of care. Overall, the schematic emphasizes that stigma and professional attitudes are embedded within a broader network of contextual and relational factors, reinforcing the importance of integrated approaches involving education, communication, organizational support, and stigma-sensitive care in healthcare settings.

## 4. Discussion

This scoping review aimed to map the available evidence on stigma and attitudes among nurses in relation to chemsex. The findings reveal a limited, heterogeneous, and still fragmented body of literature, with few studies directly addressing nursing perspectives and an even smaller number explicitly examining stigma as a central construct. Moreover, the included studies varied considerably in methodological design, evidentiary scope, and participant composition, ranging from exploratory qualitative reports and professional reflections to cross-sectional surveys involving multidisciplinary healthcare teams. Within this context, the findings should be interpreted primarily as exploratory and hypothesis-generating rather than confirmatory. Nevertheless, the synthesis identified important recurring themes and potential interconnections between knowledge, professional attitudes, clinical practice, and structural factors, offering valuable insights into the current state of the field and its implications for nursing practice and future research.

Importantly, this review goes beyond a descriptive mapping of the literature by advancing a broader understanding of how stigma operates in the context of chemsex-related care. Rather than functioning merely as a background or contextual factor, stigma appears to be an important underlying dimension that may influence nursing practice, influencing how professionals perceive, approach, and engage with individuals who engage in chemsex. This perspective shifts the focus from isolated competencies toward a more integrated understanding of how knowledge, attitudes, and structural conditions may interact to influence care delivery.

One of the most striking findings of this review is the limited availability of studies specifically focused on nurses. Although healthcare professionals are frequently included in the literature on chemsex, most studies adopt multidisciplinary perspectives, often grouping nurses together with other professional categories [10,17]. This lack of professional disaggregation limits a more nuanced understanding of how nursing-specific knowledge, attitudes, and practices may shape chemsex-related care. Nevertheless, from a nursing practice perspective, these broader multidisciplinary contexts remain highly relevant, as nurses frequently sustain longitudinal patient contact across diverse healthcare settings, including sexual health services, primary care, mental health care, and harm reduction programs. In these contexts, nursing practice is closely associated with communication, therapeutic engagement, health education, continuity of care, and coordination between services. Consequently, examining multidisciplinary evidence may contribute to a broader understanding of the clinical, relational, and organizational environments in which nursing care related to chemsex is developed and negotiated.

Another key finding relates to the way stigma is represented in the literature. Across the included studies, stigma was rarely measured directly. Instead, it emerged implicitly through descriptions of patient experiences, professional attitudes, and recommendations for non-judgmental care. This finding both confirms and extends existing theoretical frameworks of stigma in healthcare. According to Link and Phelan [13], stigma involves processes of labeling, stereotyping, and discrimination operating within power structures. The findings of this review suggest that, in the context of chemsex, stigma operates not only at the interpersonal level, through patient-provider interactions, but also structurally, through service organization, training gaps, and institutional constraints. This broader perspective aligns with the work of Parker and Aggleton [14], who emphasize the role of social and structural forces in shaping stigmatizing processes.

The absence of explicit measurement of stigma is particularly noteworthy. Without validated instruments or systematic assessment, it becomes difficult to quantify the extent of stigma among healthcare professionals or to evaluate the effectiveness of interventions aimed at reducing it. This limitation has been highlighted in other areas of healthcare, such as HIV and substance use, where the development of stigma measurement tools has been essential for advancing research and informing practice [25,26]. The lack of similar efforts in the field of chemsex suggests that this area remains in an early stage of conceptual and methodological development and highlights an urgent need for theory-driven empirical research.

Closely related to stigma are professional attitudes, which were also found to be insufficiently explored. While several studies emphasized the importance of empathy, non-judgmental communication, and patient-centered care, these aspects were typically presented as recommendations rather than empirically assessed variables [10,15]. This gap limits the understanding of how nurses perceive individuals who engage in chemsex, how comfortable they feel addressing the topic, and how their personal beliefs and values may influence care. In this sense, the findings challenge the implicit assumption that non-judgmental care is uniformly practiced, suggesting instead that such approaches may depend on underlying competencies, training, and contextual support.

The findings also highlight important gaps in knowledge and preparedness among healthcare professionals. Although some studies reported general awareness of chemsex, knowledge was often partial and accompanied by low self-perceived competence [18]. This lack of preparedness may contribute to discomfort in addressing the topic, avoidance of discussions, and missed opportunities for intervention. Importantly, this review suggests that knowledge deficits should not be viewed in isolation but as part of a broader system in which knowledge, attitudes, and stigma are mutually reinforcing.

In this regard, the schematic representation developed from the thematic synthesis helps illustrate how knowledge, stigma-related attitudes, and organizational factors may interact within chemsex-related healthcare practice. The relationships identified across the included studies suggest that limited training opportunities, fragmented service structures, and broader contextual barriers may influence professional attitudes and healthcare experiences in this field. Conversely, greater preparedness and communication competencies were frequently associated with more patient-centered and non-judgmental approaches to care. Rather than proposing a causal model, this synthesized perspective highlights the potential interconnections between individual, organizational, and structural dimensions involved in healthcare responses to chemsex, reinforcing the relevance of integrated and stigma-sensitive approaches within nursing and multidisciplinary practice.

The analysis also revealed significant variability in clinical practice. While some professionals reported routinely addressing chemsex, others did not incorporate the topic into their assessments. This inconsistency reflects the absence of standardized protocols, which has a direct impact on nursing professional autonomy. From a theoretical perspective, this variability can be interpreted as a manifestation of structural stigma, where institutional conditions shape what is considered legitimate or feasible within the scope of nursing practice [14]. Without clear clinical pathways, nurses may find their ability to provide proactive, evidence-based interventions curtailed, reducing their role to a reactive rather than a transformative presence in patient care.

Barriers to care were identified at multiple levels. At the individual level, professionals reported a lack of knowledge and discomfort. At the organizational level, barriers included limited time and fragmented services [10]. Critically, addressing these barriers is not merely a matter of technical proficiency but a central tenet of patient advocacy, a core ethical pillar of the nursing profession. Nurses have a professional mandate to act as the patient’s advocate, challenging both individual biases and structural stigma that impede equitable access to care. Addressing these barriers, therefore, requires a multi-level approach that goes beyond individual training to include organizational changes that empower nurses to fulfill their ethical role as protectors of patient dignity.

## 5. Limitations

This review has several limitations that should be considered. First, the number of included studies was small and characterized by substantial heterogeneity in design, populations, and scope. Notably, only one study focused exclusively on nurses, while the remaining studies included broader groups of healthcare professionals. As a result, some findings rely on indirect evidence, limiting the specificity of conclusions regarding nursing practice.

Second, stigma was rarely assessed as an explicit construct and was instead inferred from qualitative data and discussions of clinical practice. This limits the ability to fully capture its magnitude and to compare findings across studies, reflecting an important methodological gap in the field.

Third, the inclusion of multidisciplinary samples may have diluted nursing-specific perspectives, as it was not always possible to distinguish the contributions of different professional groups. In addition, the restriction to studies published in English, Portuguese, and Spanish may have excluded relevant evidence from other contexts.

Finally, the interpretative nature of thematic synthesis may introduce subjectivity in data analysis. Despite these limitations, this review provides a structured synthesis of an emerging and underexplored field, highlighting critical gaps and informing future research and nursing practice.

## 6. Implications for Nursing Practice

The findings of this review highlight important implications for nursing practice, particularly in relation to the care of individuals who engage in chemsex within increasingly complex, stigmatized, and multidimensional healthcare contexts. As frontline professionals working across sexual health services, primary care, emergency settings, mental health care, and harm reduction programs, nurses are well positioned in identifying vulnerabilities, facilitate access to care, and promote therapeutic engagement among populations that frequently experience marginalization and barriers within healthcare systems. However, the ability of nurses to effectively fulfill this role depends not only on technical knowledge but also on the development of relational, cultural, and organizational competencies capable of supporting stigma-informed and patient-centered care.

A central implication emerging from this review concerns the pervasive role of stigma in shaping healthcare interactions. Although stigma was rarely measured directly in the included studies, it consistently appeared through narratives related to judgment, communication difficulties, avoidance of care, and patient reluctance to disclose chemsex-related practices. This finding reinforces existing evidence demonstrating that stigma associated with substance use and sexual behavior contributes to delayed healthcare seeking, fragmented care trajectories, and poorer health outcomes, particularly among socially marginalized groups [6,26]. Within nursing practice, this underscores the importance of fostering critical awareness regarding both individual biases and broader structural forms of stigma embedded in healthcare institutions and professional cultures. Educational strategies aimed at reflective practice, bias recognition, and non-judgmental communication may therefore play a crucial role in strengthening therapeutic relationships and improving patient engagement [26].

The review also emphasizes that communication constitutes a core component of effective care in the context of chemsex. Establishing trust and creating environments in which individuals feel safe to discuss highly sensitive behaviors are essential conditions for comprehensive assessment, counseling, and continuity of care [5,6]. In this regard, nursing practice requires competencies that extend beyond interpersonal communication skills alone. Cultural sensitivity, understanding of sexual diversity, familiarity with harm reduction principles, and the ability to navigate the intersections between sexuality, substance use, and mental health are all fundamental to supporting meaningful patient-centered care. Consistent with findings from the broader sexual health and substance use literature, empathetic and affirming communication appears to be directly associated with greater disclosure, adherence to care, and improved therapeutic outcomes [12,27,28].

Another important implication relates to the significant gaps in knowledge and preparedness identified across the studies. Limited familiarity with chemsex-related issues, low confidence in addressing the topic, and inconsistencies in clinical approaches suggest that chemsex remains insufficiently integrated into nursing education and continuing professional development [18]. Importantly, this gap extends beyond the pharmacological aspects of substance use and encompasses broader psychosocial, behavioral, and structural dimensions of care. Integrating chemsex-related content into nursing curricula and professional training programs may therefore contribute not only to improving technical competence but also to reducing uncertainty, discomfort, and stigma in clinical practice. Given the complexity of chemsex-related care, educational initiatives should adopt interdisciplinary and practice-oriented approaches that incorporate harm reduction, sexual health, mental health, communication strategies, and the social determinants of health [8].

The findings further suggest that the absence of structured clinical guidance contributes to variability and inconsistency in nursing practice. Across the included studies, approaches to chemsex-related care often depended on individual professional initiative rather than standardized institutional protocols. This variability may result in missed opportunities for screening, counseling, prevention, and referral. Drawing from evidence in HIV prevention and substance use care, the implementation of structured assessment tools, brief interventions, and integrated referral pathways may strengthen the quality, consistency, and continuity of nursing care [16]. In this context, nurses may play a central role not only in direct patient care but also in coordinating interdisciplinary interventions and facilitating integration between sexual health, mental health, and addiction services.

The review additionally reinforces the relevance of adopting harm reduction as a guiding framework for nursing practice in chemsex-related care. Harm reduction approaches prioritize autonomy, safety, and pragmatic engagement with patients’ lived realities rather than moralizing or abstinence-based perspectives [1]. This perspective is particularly important in the context of chemsex, where stigmatizing attitudes may discourage disclosure and disengage individuals from healthcare services. Nurses are uniquely positioned to operationalize harm reduction strategies through counseling, sexual health education, linkage to PrEP and mental health services, safer substance use guidance, and ongoing therapeutic support. Embedding harm reduction principles into nursing practice may therefore contribute to more inclusive, responsive, and equitable models of care.

Importantly, the findings of this review also indicate that many of the barriers affecting chemsex-related care are structural rather than exclusively individual. Limited consultation time, fragmented services, insufficient institutional support, and lack of training opportunities constrain the capacity of nurses to adequately address chemsex within routine practice [5,6,11]. Consequently, strengthening nursing practice in this field requires organizational and policy-level interventions capable of supporting integrated models of care, interdisciplinary collaboration, and continuing professional education. Beyond their clinical role, nurses may also contribute to service development, advocacy, and quality improvement initiatives aimed at reducing inequities and improving healthcare accessibility for individuals who engage in chemsex.

Finally, the emotional and psychological demands associated with caring for stigmatized and socially vulnerable populations should not be overlooked. Emerging evidence suggests that healthcare professionals working in this context may experience stress, uncertainty, emotional burden, and burnout [5,11,12]. Supporting nurses through supervision, peer support, reflective spaces, and institutional strategies that promote resilience and professional well-being is therefore essential, both for sustaining the workforce and for ensuring the delivery of high-quality, compassionate care [5,6,12].

Taken together, these implications highlight the need for a comprehensive and multi-level approach to strengthening nursing practice in the context of chemsex. Addressing stigma, enhancing professional competencies, implementing harm reduction principles, developing clinical guidance, and ensuring organizational support are interconnected components of this process. By positioning nurses as key agents in the delivery of stigma-informed, culturally competent, and patient-centered care, these efforts may contribute substantially to improving health outcomes and reducing inequities among individuals who engage in chemsex.

## 7. Conclusions

This scoping review highlights an important and still insufficiently explored area in the literature concerning stigma and attitudes among nurses in the context of chemsex. Despite the growing recognition of chemsex as a complex and multifaceted public health issue, evidence specifically addressing nursing perspectives remains limited, fragmented, and largely indirect. Notably, although stigma consistently emerged as a relevant barrier to care, it was rarely examined as an explicit construct, restricting a more comprehensive understanding of how stigma-related dynamics may influence professional interactions, communication, and patient engagement.

The findings indicate potential interconnections between knowledge, professional attitudes, preparedness, and clinical practices within chemsex-related care contexts. In this sense, gaps in training, institutional support, and clinical guidance may contribute to uncertainty and inconsistencies in care delivery, including difficulties in addressing sensitive issues related to sexuality and substance use. At the same time, the findings reinforce the potential relevance of nursing practice in promoting equitable, stigma-informed, and patient-centered approaches to care.

Advancing nursing practice in the field of chemsex requires greater integration between research, education, clinical practice, and health policy. Future studies should prioritize the explicit examination of stigma and attitudes among nurses, as well as the development of validated assessment tools and educational interventions tailored to chemsex-related care. Strengthening competencies related to communication, cultural sensitivity, and harm reduction may contribute to improving therapeutic engagement and the quality of care provided to individuals who engage in chemsex.

Importantly, the limited representation of nursing-specific evidence identified in this review should not be interpreted as evidence of the limited relevance of nursing within chemsex-related care. Rather, it reflects a broader gap in the scientific literature concerning a professional group that is frequently involved in patient communication, health education, continuity of care, and harm reduction practices across multiple healthcare settings.

Ultimately, addressing chemsex in healthcare settings requires moving beyond fragmented and reactive approaches toward more comprehensive, stigma-aware, and person-centered models of care. Within this process, nursing may contribute substantially to strengthening inclusive, responsive, and therapeutically engaged healthcare practices for individuals who engage in chemsex.

## Figures and Tables

**Table 1:** Characteristics of the studies included in the scoping review.

Author/Year	Country	Study Objective	Design	Participants	Context	Main Focus
Evers et al., 2020 [23]	Netherlands	Assess the frequency, facilitators, and barriers to addressing chemsex in consultations	Cross-sectional (survey)	108 STI clinic nurses (100% nursing sample)	Sexual health clinics	Knowledge, attitudes, practice, and barriers in addressing chemsex; Instrument: Online self-administered survey questionnaire developed for the study
Carney, 2026 [15]	USA	Explore clinical management and communication in cases of patients with chemsex	Case study (case challenge)	Nurse practitioners (Advanced Practice Nurses)	Primary care	Affirmative communication, harm reduction, and barriers to care
Kingsley, 2024 [24]	United Kingdom	Describe essential knowledge about chemsex for emergency professionals	Practice-oriented professional article	EMS professionals (data aggregated within multidisciplinary teams).	Emergency services	Recognition, clinical management, and risks associated with chemsex
Hiransuthikul et al., 2025 [17]	Thailand	Identify essential components of harm reduction services in chemsex	Qualitative (interviews)	MSM, family members and healthcare professionals (data aggregated within multidisciplinary teams).	Sexual and mental health services	Service structure, stigma, and care needs
McGaughey et al., 2023 [10]	United Kingdom	Exploring stakeholder experiences in harm reduction in chemsex	Qualitative	Service professionals and managers (data aggregated within multidisciplinary teams).	Harm reduction services	Perceptions, stigma, burnout, and organization of care
Lo Moro et al., 2025 [18]	Italy	Evaluating knowledge and practices about PrEP among addiction service professionals	Cross-sectional	306 healthcare professionals (including nurses; data aggregated)	Addiction services	Knowledge, practice, training, and barriers in HIV prevention associated with chemsex; Instrument: Structured, self-administered questionnaire

Note: Abbreviations defined: EMS = Emergency Medical Services; HIV = Human Immunodeficiency Virus; MSM = Men who have Sex with Men; PrEP = Pre-Exposure Prophylaxis; STI = Sexually Transmitted Infection; USA = United States of America.

**Table 2:** Analytical extraction from the included studies.

Study	Professional	Knowledge	Attitudes/Stigma	Clinical Practice	Barriers	Training
Evers et al., 2020 [23]	Nurses (STI clinics)	Knowledge considered sufficient, but with specific gaps	Generally positive attitude, but with a need for greater preparation to address the topic	71% address chemsex during consultations	Lack of time and difficulty connecting with patients	76% report a need for training
Carney, 2026 [15]	Nurse practitioners	Not directly assessed	Emphasis on non-judgmental care; recognition of prior stigma experienced by patients	Patient-centered approach, affirmative communication, and harm reduction	Previous negative patient experiences lead to avoidance of care	Implicit training in cultural skills and communication
Kingsley, 2024 [24]	EMS professionals	Need to increase knowledge about chemsex	Emphasis on a non-judgmental, empathetic, and understanding approach	Recognition and management of chemsex-related emergencies	Lack of familiarity with the phenomenon and hidden context	Need for capacity building for an appropriate response
Hiransuthikul et al., 2025 [17]	Healthcare professionals + stakeholders	Need to improve literacy about chemsex	Structural stigma identified as a barrier to accessing care	Development of integrated harm reduction services	Stigma, discrimination, and legal barriers	Need for structured services and capacity building
McGaughey et al., 2023 [10]	Harm reduction professionals	Not central	Emphasis on an empathetic and non-judgmental approach	Continuous support and follow-up in harm reduction	Burnout, fragmentation of services, limited access	Need for institutional support and capacity building
Lo Moro et al., 2025 [18]	Healthcare professionals (including nurses)	Low level of knowledge (≈45% knew about PrEP)	Varying perceptions about competence and professional role	Low implementation of PrEP in practice	Lack of training, structural difficulties	87.9% reported a lack of training

**Table 3:** Thematic synthesis of findings on stigma and attitudes of healthcare professionals towards chemsex.

Topic	Description	Evidence from Studies	Implications for Nursing
1. Stigma as a barrier to care	Stigma (explicit or implicit) negatively influences access to and continuity of healthcare.	Patients report avoiding services due to negative experiences and judgment; structural stigma identified in services	Nurses need to recognize the impact of stigma and develop non-discriminatory care practices.
2. Need for a non-judgmental approach	Empathetic and non-judgmental attitudes are central to care in chemsex.	Emphasis on affirmative and patient-centered communication; empathy-based and supportive approach	Training in sensitive and culturally competent communication should be prioritized in nursing.
3. Knowledge gaps and professional insecurity	Professionals have limited knowledge and insecurity in addressing chemsex.	Partial knowledge and need for training; low awareness about PrEP and prevention	Nurses may avoid addressing the topic due to insecurity, reinforcing gaps in care.
4. Inconsistent clinical practice	The approach to chemsex is not systematic and depends on the professional and the context.	Not all professionals address the topic regularly; lack of standardized protocols	Need for specific clinical guidelines for nursing.
5. Need for training and capacity building	There is significant demand for specific training on chemsex.	High need for training reported; lack of structured training	Development of educational programs focused on nursing
6. Structural and organizational barriers	Institutional factors hinder the proper approach to chemsex.	Lack of time, fragmentation of services and burnout	Nurses operate in systems that limit the implementation of appropriate practices.
7. Centrality of patient-centered care and harm reduction	Effective care involves a holistic, patient-centered, and harm reduction-based approach.	Integration of strategies such as PrEP, counseling and active listening	Reinforces the strategic role of nursing in coordinating care

**Table 4:** Knowledge gaps and implications for research and practice in nursing.

Thematic Area	Current Evidence	Gap Identified	Implications for Research	Implications for Nursing Practice
Stigma among nurses	Stigma appears indirectly (patient perception, need for non-judgmental care)	Lack of studies that directly measure stigma in nurses	Develop quantitative studies with validated instruments to measure stigma	Awareness and training to reduce stigmatizing attitudes
Professional attitudes and belief	Attitudes inferred (empathy, non-judgmental approach), but not systematically evaluated	Lack of specific studies on nurses’ attitudes towards chemsex	Investigate attitudes, moral values, and professional comfort	Training in communication and culturally competent care
Knowledge about chemsex	Partial knowledge and significant gaps	Lack of standardized assessment of nursing knowledge	Develop instruments and comparative studies between professional categories	Continuing education and inclusion of the topic in nursing training
Educational interventions	Few training studies (one-off interventions)	Lack of robust trials or evaluations of educational programs	Test educational interventions (quasi-experimental, RCTs)	Implementing structured training programs
Clinical nursing practice	The approach to chemsex is inconsistent and poorly structured	Lack of specific clinical protocols for nursing	Implementation and evaluation studies of care protocols	Development of clinical guidelines and care flows
Integration of care	Fragmented services and difficulty in coordination between areas	Lack of integrated care models focused on chemsex	Investigate interdisciplinary care models	Nurses as coordinators of integrated care
Structural and organizational context	Institutional barriers (time, training, resources)	Little research on organizational impact on care	Studies at the organizational level and health policies	Need for institutional support and training policies
Neglected populations in research	Predominant focus on MSM and specialized services	Lack of studies in other contexts (primary care, general nursing)	Expand studies to different care settings	Preparing nurses at different levels of care
Mental health and emotional burden of professionals	Evidence of burnout among professionals	Little exploration of the emotional impact on nurses	Studies on emotional burden and professional coping	Implementation of psychosocial support for professionals

## Data Availability

The data supporting the findings of this study are available from the corresponding author upon reasonable request. All data analyzed during this study are included in the published article; additional information related to the study process may be obtained from the corresponding author upon reasonable request.

## Public Involvement Statement

No public or patient involvement was associated with this research.

## Guidelines and Standards Statement

This study was conducted and reported in accordance with the Joanna Briggs Institute (JBI) methodology for scoping reviews and the Preferred Reporting Items for Systematic Reviews and Meta-Analyses extension for Scoping Reviews (PRISMA-ScR).

## Appendix A

**Table A1:** Detailed search strategies for all databases.

Database	Search Strategy	Search Fields	Date of Search	Records Retrieved (n)
PubMed/MEDLINE	(“chemsex” OR “sexualized drug use” OR “sexualised drug use” OR “party and play” OR “PnP” OR “drug use during sex” OR “slamming” OR “wired play” OR “high and horny” OR “chemplay” OR “boosting”) AND (nurse* OR nursing OR “healthcare professional*” OR “health care provider*” OR clinician* OR “health personnel” OR “sexual health staff” OR “harm reduction staff”) AND (stigma OR stigmatization OR prejudice OR discrimination OR bias OR attitude OR attitudes OR perception* OR communication OR preparedness)	Title/Abstract + MeSH Terms	20 May 2026	679
Scopus	TITLE-ABS-KEY (chemsex OR “sexualized drug use” OR “sexualised drug use” OR “party and play” OR “PnP” OR “drug use during sex” OR “slamming” OR “wired play” OR “high and horny” OR “chemplay” OR “boosting”) AND TITLE-ABS-KEY (nurse* OR nursing OR “healthcare professional*” OR clinician* OR “health personnel” OR “sexual health staff” OR “harm reduction staff”) AND TITLE-ABS-KEY (stigma OR stigmatization OR prejudice OR discrimination OR bias OR attitude OR attitudes OR perception* OR communication OR preparedness)	Title, Abstract, Keywords	20 May 2026	510
Web of Science	TS = (chemsex OR “sexualized drug use” OR “sexualised drug use” OR “party and play” OR “PnP” OR “drug use during sex” OR “slamming” OR “wired play” OR “high and horny” OR “chemplay” OR “boosting”) AND TS = (nurse* OR nursing OR “healthcare professional*” OR clinician* OR “health personnel” OR “sexual health staff” OR “harm reduction staff”) AND TS = (stigma OR stigmatization OR prejudice OR discrimination OR bias OR attitude OR attitudes OR perception* OR communication OR preparedness)	Topic (Title, Abstract, Keywords)	20 May 2026	523
CINAHL (EBSCOhost)	((TI chemsex OR AB chemsex OR TI “sexualized drug use” OR AB “sexualized drug use” OR TI “sexualised drug use” OR AB “sexualised drug use” OR TI “party and play” OR AB “party and play” OR TI PnP OR AB PnP OR TI slamming OR AB slamming OR TI chemplay OR AB chemplay OR TI “wired play” OR AB “wired play” OR TI “high and horny” OR AB “high and horny”)) AND ((TI nurse* OR AB nurse* OR TI nursing OR AB nursing OR TI “healthcare professional*” OR AB “healthcare professional*” OR TI clinician* OR AB clinician* OR TI “health personnel” OR AB “health personnel”)) AND ((TI stigma OR AB stigma OR TI stigmatization OR AB stigmatization OR TI prejudice OR AB prejudice OR TI discrimination OR AB discrimination OR TI bias OR AB bias OR TI attitude* OR AB attitude*))	Title/Abstract + CINAHL Headings	20 May 2026	52
EMBASE	(‘chemsex’:ti,ab,kw OR ‘sexualized drug use’:ti,ab,kw OR ‘sexualised drug use’:ti,ab,kw OR ‘party and play’:ti,ab,kw OR ‘PnP’:ti,ab,kw OR ‘drug use during sex’:ti,ab,kw OR ‘slamming’:ti,ab,kw OR ‘wired play’:ti,ab,kw OR ‘high and horny’:ti,ab,kw OR ‘chemplay’:ti,ab,kw OR ‘boosting’:ti,ab,kw) AND (‘nurse’:ti,ab,kw OR ‘nurses’:ti,ab,kw OR ‘nursing’:ti,ab,kw OR ‘healthcare professional’:ti,ab,kw OR ‘health personnel’:ti,ab,kw OR ‘clinician’:ti,ab,kw OR ‘sexual health staff’:ti,ab,kw OR ‘harm reduction staff’:ti,ab,kw) AND (‘stigma’:ti,ab,kw OR ‘stigmatization’:ti,ab,kw OR ‘prejudice’:ti,ab,kw OR ‘discrimination’:ti,ab,kw OR ‘bias’:ti,ab,kw OR ‘attitude’:ti,ab,kw OR ‘attitudes’:ti,ab,kw OR ‘perception’:ti,ab,kw OR ‘communication’:ti,ab,kw OR ‘preparedness’:ti,ab,kw)	Title/Abstract/Keywords + Emtree Terms	20 May 2026	188
LILACS (BVS)	(tw:(chemsex OR “sexualized drug use” OR “sexualised drug use” OR “party and play” OR “uso sexualizado de drogas” OR “uso de drogas em contexto sexual” OR “slamming” OR “wired play” OR “high and horny” OR “chemplay” OR “boosting”)) AND (tw:(nurse* OR nursing OR enfermagem OR enfermeiro* OR enfermera* OR “healthcare professional*” OR “profissionais de saúde” OR “personal de salud”)) AND (tw:(stigma OR estigma OR stigmatization OR preconceito OR discriminação OR discriminación OR bias OR attitude* OR atitude* OR actitud* OR perception* OR comunicação OR comunicación OR preparedness))	Title/Abstract/Subject Terms	20 May 2026	160
Google Scholar (Grey Literature)	(“chemsex” OR “sexualized drug use” OR “sexualised drug use” OR “party and play” OR “PnP” OR “slamming” OR “wired play” OR “high and horny” OR “chemplay” OR “boosting”) AND (“nurse*” OR “nursing” OR “healthcare professionals” OR clinicians OR “health personnel”) AND (“stigma” OR “attitudes” OR “prejudice” OR “discrimination” OR “bias” OR “communication” OR “preparedness”)	Full text (first 200 results sorted by relevance)	20 May 2026	200
